# Blue light insertion at night is involved in sleep and arousal-promoting response delays and depressive-like emotion in mice

**DOI:** 10.1042/BSR20204033

**Published:** 2021-03-04

**Authors:** Fan Wu, Shuo Wu, Qiuqi Gui, Kaixin Tang, Qiqi Xu, Yue Tao, Meixuan Chen, Juan Cheng, Liecheng Wang, Lesha Zhang

**Affiliations:** Department of Physiology, School of Basic Medical Sciences, Anhui Medical University, Hefei, China

**Keywords:** Arousal, Blue Light, Brain Regions, Circadian Rhythm, Depressive-like Emotion, Sleep

## Abstract

Light plays a direct crucial role in the switch between sleep and arousal and the regulation of physiology and behaviour, such as circadian rhythms and emotional change. Artificial lights, which are different from natural light sources with a continuous light spectrum, are composed of three single-colour lights and are increasingly applied in modern society. However, *in vivo* research on the mechanisms of blue light-regulated sleep and arousal is still insufficient. In this work, we detected the effects of inserting white or blue light for 1 h during the dark period on the wheel-running activity and sucrose preference of C57 mice. The results showed that blue light could induce delays in sleep and arousal-promoting responses. Furthermore, this lighting pattern, including blue light alone, induced depressive-like emotions. The c-fos expression in the blue light group was significantly higher in the arcuate hypothalamic nucleus (Arc) and significantly lower in the cingulate cortex (Cg) and anterior part of the paraventricular thalamic nucleus (PVA) than in the white light group. Compared with the white light group, the phospho-ERK expression in the paraventricular hypothalamic nucleus (PVN) and PVA was lower in the blue light group. These molecular changes indicated that certain brain regions are involved in blue light-induced response processes. This study may provide useful information to explore the specific mechanism of special light-regulated physiological function.

## Introduction

Sleep and arousal are bioregulated by inner homeostasis and circadian rhythm [1]. Light plays a direct crucial role in the switch between sleep and arousal [2], but its mechanism is not fully understood. Currently, many professions conduct shift work or work across multiple time zones, which leads to the nonconformity between environmental light and circadian rhythm and furthers intermittent sleep disorder [3,4], inducing various physiological and functional diseases [5,6]. Additionally, abnormal sleep/arousal switching could lead to delayed sleep–wake phase disorder [7]. Therefore, it is critical to explore the specific mechanism of light-regulated sleep and awakening to find effective treatment.

Furthermore, during the recent decade, man-made illuminant light-emitting diodes (LEDs) have been increasingly applied in electrical equipment, such as TV screens, computer monitors and cell phones. Additionally, the public spends gradually more time gazing at LED screens, leading to the emerging public health problem of digital eye strain issues [8]. White LED light sources, which are different from the natural light sources of the continuous light spectrum, are composed of multicoloured light of red, green and blue. The short and medium waves of blue light, where the wavelength is between 400 nm and 440 nm, produce high energy. This kind of energy can enhance the toxic accumulation in the macular area in eyes, threatening the health of fundus oculi [9] and inducing cellular apoptosis [10], in addition to the generation of reactive oxygen species in skin [11]. In contrast, blue light of 480 nm wavelength can effectively stimulate melanopsin in the intrinsically photosensitive retinal ganglion cells (ipRGCs) of the retina, which contributes to circadian rhythm regulation [12]. Researchers have indicated that potential health hazards that are due to artificial blue light are growing in modern ageing society [13]. Heo et al. showed that the use of blue light LED smartphones at night may negatively influence sleep [14]. However, it is unclear whether exposure to excessive long-wavelength blue light could induce sleep/arousal and circadian rhythm changes. Consequently, studying how light, especially blue light, influences sleep can supply scientific evidence for helping to improve sleep.

Photosensitive retinal cells can transform the information of light to electrical signals and then project to distinct brain regions through the emerging visual sense of bipolar cells and optic ganglion cells. The lights received by ipRGCs are transformed into nerve impulses and projected to broad target nuclei, primarily the suprachiasmatic nuclei (SCN), ventrolateral preoptic area (VLPO) and nuclei related to non-image-forming (NIF) functions involving regulation of circadian rhythm, pupillary light reflex, sleep and arousal cycles, and other physiological functions [15–18]. It has been reported that light of distinct wavelengths shows diverse influences on mammalian sleep/awake cycles, among which blue light could delay the onset of sleep or awake [18]. Esaki’s group has indicated that wearing amber glasses that block blue light could help subjects with delayed sleep phase disorder (DSPD) significantly advance sleep onset time [19]. However, the specific mechanism remains unknown.

Furthermore, irregular light exposure may induce both circadian rhythm disorder and mood change, causing negative emotions, such as depression and anxiety, and even cognitive function deficits [20]. For example, under short photoperiod mimicking the development of seasonal affective disorder, rodents may develop depression- and anxiety-like behaviour, which could be relieved by exposure to either intense wide-spectrum white light or to blue light [21]. It has been reported that artificial light sources, including blue light, may disturb circadian rhythm and further lead to emotional change [22]. However, it is controversial whether blue light exposure could cause depression. A few reports have indicated that blue-light deprivation induces depression-like behaviour [23,24]. Lyilikci’s group showed that blue light stimulation in the dark had an antidepressant effect [25]. Another group presented that white light that was filtered through the blue light wavelength portion induced depression-like responses in rats, and temporary spatial learning deficits could be detected [24].

Downstream molecular signalling of blue light-induced responses has been studied in plants or nonmammalian animals [26–28]. However, evidence of the molecular mechanism in the brain *in vivo* under blue light-regulated sleep and arousal is still insufficient. In the present study, we investigated the brain regions that relate to blue light-induced delays in sleep onset and arousal onset to provide potential active or suppressed brain region candidates. We acquired cryostat sections of the whole brains of mice that had experienced a 1-week period of blue or white light stimulation 1 h per day, and we then detected and compared the level of the immediate early gene c-fos and MAPK pathway downstream signal molecule phosphorylated ERK activity using immunohistochemistry.

## Materials and methods

### Animal treatment

**Figure 1:**
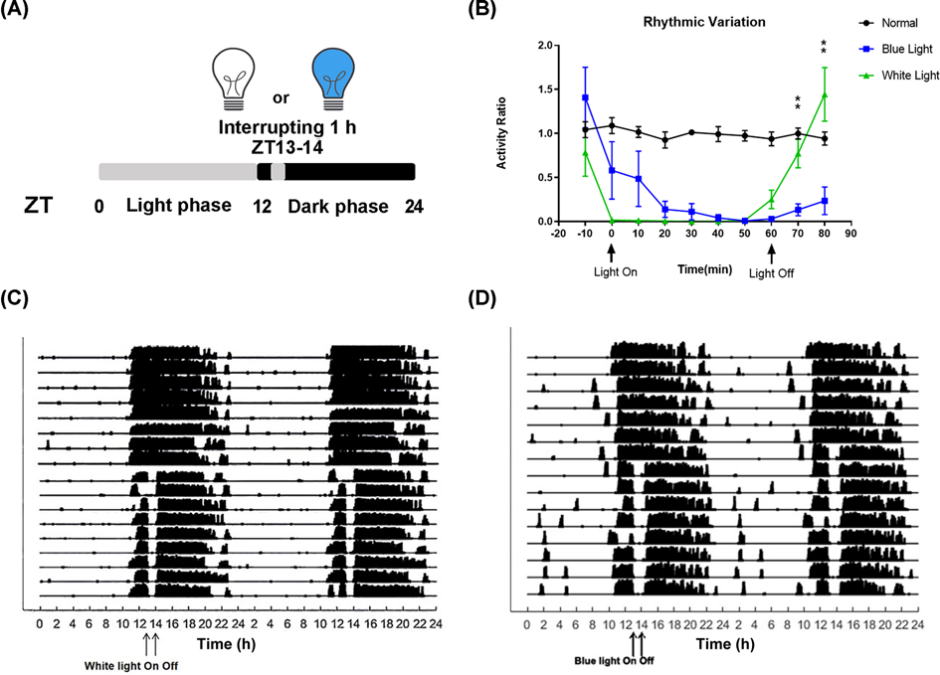
Blue light induces delays in both sleep and arousal-promoting responses (**A**) Inserted white and blue illumination (470 nm, 720 lux) patterns for wheel-running recording. (**B**) The activity ratios of four mice in each group (normal group, white light group and blue light group) during the middle five days were used for statistics. Except for the normal group, mice were exposed to white light or blue light for 1 h at ZT13 (0–60 min). Activity ratios are shown as the MEAN ± SEM. One-way repeated measures ANOVA for light condition during the first 10 min after finishing light exposure, *F*_(2,9)_ = 17.29, *P*=0.0008. Posthoc Tukey blue versus white **P<0.01. One-way repeated measures ANOVA for light condition during the first 20 min after finishing light exposure, *F*_(2,9)_ = 8.991, *P*=0.0071. Posthoc Tukey blue versus white **P<0.01. (**C**) Actogram of wheel running of mouse housed under a normal circadian rhythm of 12-h light/12-h dark for 7 days switched to a 12-h light/12-h dark cycle with a 1 h white light (C) or blue light (D) disturbance at ZT13-14 for 7 days.

**Figure 2:**
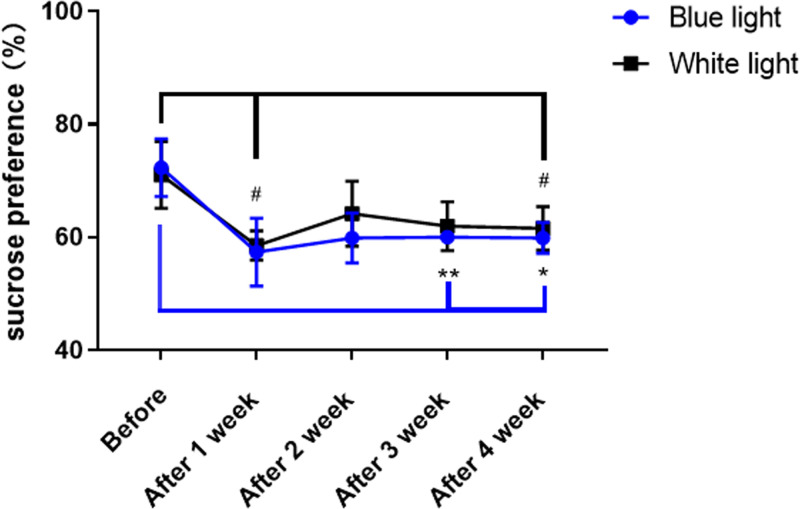
Sucrose preference test Mice exposed to white or blue light for 1 h at ZT13 exhibited disturbances for four continuous weeks, with both decreased sucrose preference scores. The sucrose preference ratio is shown as the MEAN ± SEM. Differences between 3 weeks after 1 h of blue light interference at ZT13 and normal circadian rhythm (*t*_(3)_ = 5.988, **P<0.01) and 4 weeks (*t*_(3)_ = 5.695, *P<0.05) after 1 h of blue light interference at ZT13 and normal circadian rhythm. Differences between 1 week (*t*_(3)_ = 3.191, ^#^*P*<0.05) or 4 weeks (*t*_(3)_ = 3.58, ^#^*P*<0.05) after 1 h of white light interference at ZT13 and normal circadian rhythm. The number of mice in each group was four.

C57BL/6 mice weighing 18–20 g were housed under a 12-h light/12-h dark cycle. To avoid the individual difference induced by sexual hormones and menstrual cycles of female mice, the genders of all the mice used were male. Experiments were started after the circadian rhythm of all the mice had synchronized to this light/dark cycle. All the animals were acclimatised for at least one week. Food and water were available ad libitum at room temperature (∼25°C). All studies involving animals were reported in accordance with the ARRIVE guidelines for experiments involving animals and were carried out in accordance with the U.K. Animals (Scientific Procedures) Act, 1986 and associated guidelines, EU Directive 2010/63/EU for animal experiments, or the National Institutes of Health Guide for the Care and Use of Laboratory Animals (NIH Publications No. 8023, revised 1978). All experiments were approved by the Administration Office of Laboratory Animals of Anhui Medical University and complied with the guidelines of the Institutional Animal Care Unit Committee of Anhui Medical University. All animal experimental procedures were also permitted by the Animal Ethics Committee of Anhui Medical University (Licence No. LLSC20190117). For the circadian rhythm experiment (Figure 1), the mice of white light group and blue light group were exposed to 1 h white or blue light insertion during ZT13-ZT14, that is beginning at one hour after dark phase and lasting for one hour. The number of mice in both the white group and the blue group was four. After one-week wheel-running recording under 1 h light insertion condition, those eight mice in white light group and blue light group were killed to be detected c-fos and pERK expression by immunohistochemistry staining assay. Because of staining occasionally failed, the number of mice used for statistics in the results of immunohistochemistry experiment was no more than four in each group. For Figure 2, another eight acclimatised mice were divided into two groups, which were white light group and blue light group, being exposed to 1 h white or blue light insertion during ZT13-ZT14 and used for depressive-like emotion detection

### Light conditions

LED light bands were mounted around the inner walls of the circadian rhythm recording boxes. By testing with an illuminance meter (Unit, Guangdong, China), the wavelength of the blue LED light was verified as 470 nm, and the illumination intensity was verified as 720 Lux. Meanwhile, the white LED light source was set at the same luminance as that of blue light. The illumination intensity during the dark period was 0 Lux. The wavelength spectrum of each kind of light was showed in Supplementary Figure S1. The wavelength spectrum analyser is FLAME-S-VIS-NIR-ES, which can detect the range of 350–1000 nm (FLMS01429, Ocean Optics, U.S.A.).

### Wheel-running

Before stimulation by the insertion of 1 h blue or white light, all the animals were kept under 12-h light/12-h dark cycles with the light turned on at 11:00 a.m (ZT0). to acclimatize for at least 2 weeks. Four mice were individually housed in single cages within a larger circadian recording cage equipped with running wheels for each of them to monitor their locomotor activities, and animals were given ad libitum access to food and water. The individual cages for each mouse were made of transparent acrylic glass boards, so mice could see each other. On the outside of each of the acrylic glass cages, a magnetic sensor was installed to merge induction with another sensor on the wheel. Once the wheel is forced to move around, the wheel-running activity data were collected by a number counter recording every second and then analysed with custom-written software using MATLAB (MathWorks, Natick, MA). The amount of wheel running activity was calculated as the total number of wheel revolutions in a time period of 10 min. To eliminate individual activity differences in the mice, the activity levels of individual mice are shown as the ratio of wheel running counting numbers in a certain time period divided by the average counting numbers of the whole evaluated time period.

### Sucrose preference test

The sucrose preference test was performed as described in previous studies [29]. Eight mice were randomly separated into two groups: the white light group and the blue light group. At 11 a.m., when the light was turned off, mice in the circadian cages were separately provided with two bottles of water for 24 h, of which one contained 1.2% sucrose. Both sucrose solution and water intake volume were measured by the reduced weight method. The bottle position was changed every day to prevent location preference. Finally, the sucrose preference ratio was calculated with the following formula: [sucrose solution volume / (sucrose solution volume + water volume)] × 100%. Before receiving interrupted light (under normal 12-h light/12-h dark cycles), sucrose preference index of the mice divided into blue or white group was detected and recorded as ‘before’. Then after every one week of receiving 1 h light insertion, the sucrose preference index of the mice in those two groups were detected till one month.

### Immunohistochemistry

Ninety minutes after 1 h of insertion of white or blue light, the mice were quickly blinded and anaesthetized with sodium pentobarbital (55 mg/kg, i.p.). Deeply anaesthetized mice were punctured into their left ventricle and perfused with ice-cold saline and then 4% cold paraformaldehyde in 0.1 mol/l phosphate buffer. Brains were removed and postfixed in 4% paraformaldehyde overnight at 4°C. All the brains were transferred to and kept in a solution of 30% sucrose from the next day until they sunk. Cryostat sections of brains were cut at a thickness of 30 μm on a Leica CM3050S cryostat (Leica, Wetzlar, Germany). Brain sections were stored in 0.05% ProClin™ 300 (48912-U, Sigma-Aldrich, Germany) in 0.1 mol/l phosphate buffer at 4°C for further processing. Brain slices were washed in 0.1 mol/l phosphate buffer and then blocked with 5% BSA solution for 2 h at room temperature. Incubation with primary antibodies against c-fos (ABE457, Merck-Millipore, Germany) or pERK (9101S, Cell Signaling Technology, U.S.A.) was conducted overnight (1:100 dilution in 5% BSA solution) at 4°C. On the second day, sections were incubated with secondary antibody (biotin-conjugated AffiniPure goat anti-rabbit, 1:200 dilution in 5% BSA solution) for 2 h at room temperature. The c-fos- or pERK-positive sites were visualized using a SABC Kit and a DAB Kit. The brain sections were subsequently dehydrated in alcohol and xylene and mounted on slides under coverslips. Finally, slides were imaged on an Axio Observer 3 microscope (Carl Zeiss AG, Germany). To determine the level of c-fos and phosphorylated ERK activity, the number of stained cells in different brain regions was manually counted within the identical size area of each picture, the positive staining intensity was analysed by ImageJ of version 1.52a with the IHC toolbox plugin as well (Wayne Rasband, National Institutes of Health, U.S.A.).

### Data analysis and statistics

The results are presented as the mean ± SEM. Statistical analysis was performed using GraphPad Prism v8.0.2 (GraphPad Software, San Diego, CA, U.S.A.). Assessment of the relative activity ratio under blue LED light of 470 nm wavelength and full-wavelength white light was performed using the previously published rodent toolbox [30]. Significance of group comparisons was tested with one-way ANOVA followed with Tukey’s multiple comparisons test. In the immunohistochemistry experiment, to determine the level of c-fos or ERK activity, the numbers of stained cells in corresponding brain regions were manually counted. The data represent the mean ± SEM; significance was determined by Student’s unpaired two-tailed *t*-test. The number of animals in each group was three or four.

## Results

### Blue light induces delays in sleep and arousal-promoting responses

Studies have shown that the maximum absorption peaks of the three kinds of photosensitive opsin are 498 nm, which is the peak of rod opsin [31], 508 nm, which is the peak of M-cone opsin [32], and 470 nm, which is mainly absorbed by melanopsin [12]. Because melanopsin has been confirmed to be involved in the regulation of circadian rhythm, 1 h light exposure to blue light (470 nm) or white light (full-spectrum wavelength) was carried out to observe the sleep/arousal response in C57BL/6 mice. From the wavelength spectrum wavelength of white light, the spectrum of the white light is continuously full-wavelength and mainly includes two wave crests, of which the peaks are at 453 and 553 nm, respectively. While according to the wavelength spectrum wavelength of blue light, there is only one peak at 470 nm (Supplementary Figure S1). Furthermore, the 1 h light exposure was performed at ZT13 when animals had just aroused for 1 h with low sleep pressure. Thus, the mice were separated into two groups that received 1 h of white light (full-wavelength) or blue light (wavelength peak is 470 nm) (Figure 1A). The effects of 1 h light exposure on sleep and arousal are shown in Figure 1B. The activity ratios of four mice in the white light group and blue light group during the middle 5 days were used for statistics. Here, we randomly picked the activity ratios of four mice under normal 12-h light/ 12-h dark condition as the additional normal group to show the average active status without light insertion. The average activity ratio of the normal group was set as 1.0 and then all the activity ratios were normalized according to the average activity ratio of the normal group. Compared with white light, blue light caused delays both in sleep onset and arousal onset. In the white light group, once the light was turned on, mice quickly ceased the wheel-running activity, indicating that the switch from arousal to sleep is sharp. In the blue light group, after the light was turned on, the wheel-running activity ratio of mice decreased to one-third of that during arousal and gradually ceased for more than 20 min. Furthermore, after the white light was turned off, the mice rapidly returned to a running situation, especially during the first and second 10 min after finishing 1 h of light exposure, and the activity ratio increased significantly, showing a significant group difference when compared with the blue light group. Specifically, compared activity ratio of mice in the blue light group to that of mice in the white light group during the first 10 min after finishing light exposure, the results of one-way repeated measures ANOVA for light condition showed *F*_(2,9)_ = 17.29, *P*=0.0008. Multiple Tukey’s comparisons results showed that blue versus white *P*=0.006. Comparing activity ratio of mice during the first 20 min after finishing light exposure, we found that the activity ratio of mice in the blue light group significantly was higher than that of mice in the white group. One-way repeated measures ANOVA showed *F*_(2,9)_ = 8.991, *P*=0.0071. Multiple Tukey’s comparisons results showed that blue versus white *P*=0.0057. Furthermore, whether given white or blue light, animals exhibited normal locomotor activity during 12-h light/12-h dark cycles and reduced locomotor activity during light insertion (Figure 1C,D).

### Inserted light disturbance but not blue light contributes to depressive-like emotion

Because of the irregular exposure to light, emotional change, especially negative emotions, such as depression, could be evoked [20]. However, whether blue light exposure could cause depression is controversial. To answer this question, we then detected the sucrose preference index of mice that had been constantly exposed to either blue or white light for one month. On the detection day of each week, individual mice in the circadian cages were separately provided with two bottles of water, of which one contained 1.2% sucrose, for 24 h of free drinking. However, if we compared the sucrose preference index each week after exposure to 1 h light to the level before receiving interrupted light (under normal 12-h light/12-h dark cycles), we found that either inserting 1 h of white or blue light could result in sucrose preference reduction showing depressive-like mood, but no significant difference existed between the two groups analysed by two-way ANOVA (light condition interaction *F*_(4,24)_ = 0.4112, *P*=0.7988). However, if we compared each week after exposure to 1 h light to the level before with paired *t*-test, we found that both 1 h white light and 1 h blue light could induce a decrease in the sucrose preference index. Specifically, using paired *t*-test analysis, we found that 3 weeks (*t*_(3)_ = 5.988, *P*=0.0093) or 4 weeks (*t*_(3)_ = 5.695, *P*=0.0107) of exposure to blue light inserted for 1 h caused the sucrose preference index to be significantly lower than before; additionally, when the mice received white light inserted for 1 week (*t*_(3)_ = 3.191, *P*=0.0497) or 4 weeks (*t*_(3)_ = 3.58, *P*=0.0373) for 1 h, the sucrose preference index was lower than before (Figure 2). These results indicate that inserting either blue or white light could cause depression, which may be attributed to the interrupted light-induced potential influence on circadian rhythm and sleep.

### Several brain regions involved blue light-induced responses

**Figure 3:**
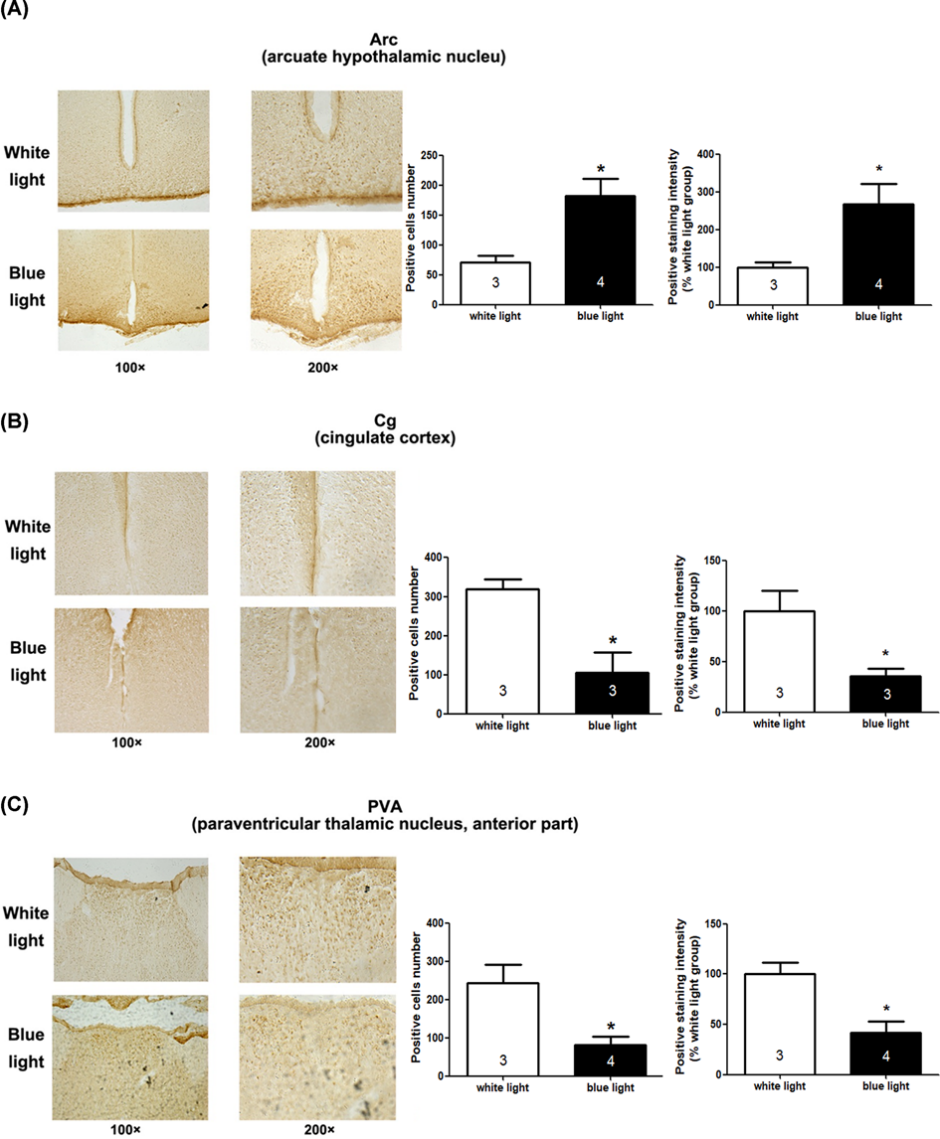
c-Fos expression in brain regions of mice exposed to white light or blue light c-Fos labelling of the arcuate hypothalamic nucleus (Arc) (**A**), cingulate cortex (Cg) (**B**) and anterior part of the paraventricular thalamic nucleus (PVA) (**C**). Positive cell numbers and positive staining intensity were analysed by unpaired *t*-test, showing that compared with the white light-treated mice, blue light induced significant changes in c-fos activation at Arc Cg and PVA (*P<0.05). The indicated number inside the column shows the number of mice calculated in each group.

**Figure 4:**
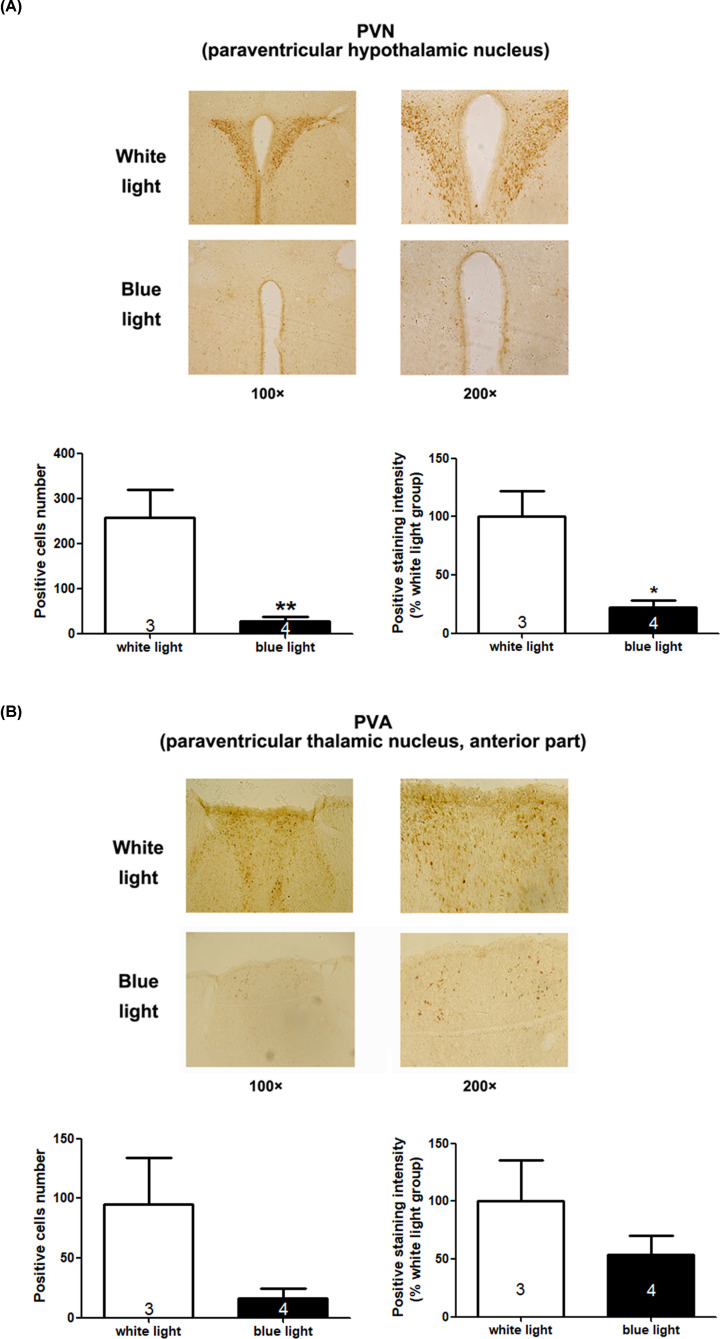
Phospho-ERK expression in brain regions of mice exposed to white or blue light Phospho-ERK labelling of the paraventricular hypothalamic nucleus (PVN) (**A**) and the anterior part of the paraventricular thalamic nucleus (PVA) (**B**). Positive cell numbers and positive staining intensity were analysed by unpaired *t*-test, showing that blue light induced a significant decrease in ERK activation in the PVN compared with white light (*P<0.05, **P<0.01). The indicated number inside the column shows the number of mice calculated in each group.

Next, to investigate the regulatory brain areas involved in this process, we investigated the protein expression levels of the immediate early gene c-fos, which is used to detect early active neural cells, and phospho-ERK (phosphorylated extracellular regulated protein kinases), which is the activated MAPK downstream signal, within the whole brains of mice in the blue light or white light exposure groups. The positive staining intensity analysis showed similar trend compared with that of positive cells number analysis. The statistical results of the immunohistochemistry assay show that c-fos positively stained cells number in the blue light group was significantly higher than that in the white light group in the arcuate hypothalamic nucleus (Arc) (positive cells number analysis: *t*_(5)_= 3.09, *P*=0.0272; positive staining intensity analysis (shown as the percentage of white light group): *t*_(5)_= 2.616, *P*=0.0473, shown in Figure 3A), but it was significantly lower than that in the white light group in the cingulate cortex (Cg) (positive cells number analysis: *t*_(4)_= 3.73, *P*=0.0203; positive staining intensity analysis: *t*_(4)_= 3, *P*=0.0399, Figure 3B) and the anterior part of the paraventricular thalamic nucleus (PVA) (positive cells number analysis: *t*_(5)_= 3.382, *P*=0.0196; positive staining intensity analysis: *t*_(5)_= 3.568, *P*=0.0161, Figure 3C). In addition, pERK expression in the blue light group was lower than that in the white light group in the paraventricular hypothalamic nucleus (PVN) (positive cells number analysis: *t*_(5)_= 4.312, *P*=0.0076; positive staining intensity analysis: *t*_(5)_= 3.977, *P*=0.0106, Figure 4A) and PVA (positive cells number analysis: *t*_(5)_= 2.309, *P*=0.069; positive staining intensity analysis: *t*_(5)_= 1.291, *P*=0.2532, Figure 4B), but the latter was not significantly different.

## Discussion

In this work, we showed that compared with white light, blue light would cause delays in both sleep onset and arousal onset. We also demonstrated that either blue or white inserted light disturbances contribute to the evocation of depressive-like emotions. Moreover, further research shows that c-fos expression in the blue light group was significantly higher than that in the white light group in the arcuate hypothalamic nucleus (Arc) but was significantly lower than that in the white light group in the cingulate cortex (Cg) and anterior part of the paraventricular thalamic nucleus (PVA). Furthermore, pERK expression in the blue light group was lower than that in the white light group in the paraventricular hypothalamic nucleus (PVN) and PVA. The changes in activated c-fos and phosphorylated ERK indicate possible brain region candidates involved in the process of blue light-induced responses.

Although light is widely known to play a critical environmental role in regulating sleep and the circadian rhythm of mammals, its physiological mechanism has not been fully researched. With the increasing application of artificial lights, their effects on sleep and circadian rhythm have attracted the attention of the public, especially the modern white LED illuminant which mimic the natural light of continuous spectrum. In the present study, from the wavelength spectrum wavelength of white light, the spectrum of the white light was nearly continuous full-wavelength but mainly included two wave crests, of which the peaks are at 455 and 555 nm respectively (Supplementary Figure S1). Moreover, the long-wavelength blue light of 480 nm, which can effectively stimulate melanopsin in ipRGCs of the retina, shows a non-imaging function involving circadian rhythm regulation. The wavelength spectrum of blue light we used included only one peak at 470 nm (Supplementary Figure S1). To mimic the intermittent contact with artificial lights at night, we chose to insert a 1 h exposure of light during the relatively active period with a low sleep desire of mice, the pattern of which was different from previous lighting patterns used to study the T7 circle, jet lag, phase shift and shortened light exposure [33]. Our results show that 1 h of exposure to blue light, but not white light, can cause nearly 20-min delays in the switch between sleep and arousal. In the report of Pilorz and her colleague, monochromatic violet light caused slight delayed sleep onset, contrarily the green light led to rapid sleep onset [34]. However, in our study, it was interesting that the white light included various light of distinct wavelengths but did not show the delayed response. Thus, further research may extend to the phenomenon when excluding of blue light from white light by light filter or transgenic mice lack of specific photoreceptor. Although the luminances of white and blue light were set at the same value, the intensities of different-wavelength light were not equal due to the visual acuity discrepancy. The 720-lux white light of the maximum wavelength at 555 nm supply an irradiance intensity of 0.10542 mW/cm^2^. The single-color blue light of 720 lux approximately output irradiance intensity of 1.158 mW/cm^2^ [35]. Therefore, the different circadian resetting response to white light and monochromatic blue light might comprehensively depends on the intensity and wavelength of lights [36].

Additionally, a previous study showed that jet lag may aggravate existing psychotic conditions by disrupting biological rhythm and probably resulting in sleep deprivation [37]. Shift work may weaken alertness and blunt performance because of sleep loss and circadian rhythm dislocation [38]. However, exposure to either 1 h of white light or blue light cannot disturb the overall sleep/arousal rhythm within 24 h light exposure, and it may not cause sleep deprivation but may induce delayed sleep and arousal-promoting responses (Figure 1). Interestingly, this kind of light pattern with a slight influence on sleep/arousal rhythm can result in depressive-like emotion. On the other hand, blue light may enhance the alertness and nervousness of the body to antagonize depression [23,24]. In the clinic, blue light exposure procedures are used in the treatment of patients with emotional disorders. For instance, blue-blocking therapy may be useful for mania in the treatment of bipolar disorder [39]. Narrow bandwidth blue light showed beneficial effects among light therapies for seasonal affective disorder [40]. Nevertheless, our results show that either blue- or white-inserted light exposure can induce emotional change because there is no difference between the sucrose preference index of the blue- and white-light inserted groups. Similarly, Xue’s laboratory has recently reported that animals that were exposed to light-at-night of 2 h blue light applied between ZT1 and ZT3 exhibited depressive-like phenotypes, but animals that were exposed to identical blue light in the daytime did not [30]. These results may be because the exposure time to blue light was short and insufficient to reverse light-induced depressive-like emotional change. This controversy should be discussed further.

In the investigation of the regulatory brain areas involved in the process of blue light-induced responses, we detected the protein expression levels of the immediate early gene c-fos and MAPK downstream signal phospho-ERK by immunohistochemistry of the whole brain. We found that the arcuate hypothalamic nucleus (Arc), cingulate cortex (Cg), anterior part of the paraventricular thalamic nucleus (PVA) and paraventricular hypothalamic nucleus (PVN) may be involved. The previous study showed that blue light could evoke *Fos* expression increase in mRNA level in the SCN and adrenal gland, but a greater *Fos* induction response to the green light than blue light in the sleep-promoting ventrolateral preoptic area (VLPO) [34]. When using optical filters removing short-wavelengths < 500 nm from polychromatic white light, phase-delay shifts was attenuated in rats and a significant reduction of c-fos activation in SCN was observed [41]. Down-regulation of c-fos was also observed in retina response to blue light deprivation in Mongolian Gerbils [23]. It has been reported that changes in neuronal activity in the lateral hypothalamus, the paraventricular nucleus and the arcuate nucleus were found after 6 h of sleep deprivation in adult male rats, which could last for up to 48 h [42]. The paraventricular thalamic nucleus has connections with the regulation of stress and negative emotional behaviour [43]. Moreover, most of the light-induced activation of multiple nuclei in the hypothalamus (paraventricular, dorsomedial and lateral hypothalamus), thalamus (paraventricular and centromedian thalamus) and limbic system is associated with ipRGCs [44]. Thus, further study could focus on those brain area candidates, and relative nervous circles relating to ipRGCs could be the key points. Therefore, in summary, the present study may provide useful information to explore the specific mechanism of special light-regulated sleep and awakening.

## Conclusions

Light plays a direct crucial role in the switch between sleep and arousal. Blue light could induce delays in sleep and arousal-promoting responses. Inserted either blue light or white light in night induced depressive-like emotion. c-fos and phospho-ERK expression changes indicated that certain brain regions are involved in the process of blue light-induced responses.

## Supplementary Material

Supplementary Figure S1

Supplementary Raw Data

## Data Availability

All the raw data have been uploaded as supplementary files including all the data analysed using GraphPad Prism v8.0.2.

## Abbreviations

Arc: arcuate hypothalamic nucleus
Cg: cingulate cortex
DSPD: delayed sleep phase disorder
ipRGCs: intrinsi cally photosensitive retinal ganglion cells
LED: light-emitting diode
NIF: non-image-forming
PVA: paraventricular thalamic nucleus
PVN: paraventricular hypothalamic nucleus
SCN: suprachiasmatic nuclei
VLPO: ventrolateral preoptic area
