# Supplementary material for: Blue light insertion at night is involved in sleep and arousal-promoting response delays and depressive-like emotion in mice
Source: Biosci Rep. 2021 Mar 4;41(3):BSR20204033. doi: 10.1042/BSR20204033 (PMC7938454; doi:10.1042/BSR20204033)
Supplement: Supplementary Figure S1 [file BSR-2020-4033_supp.pdf]

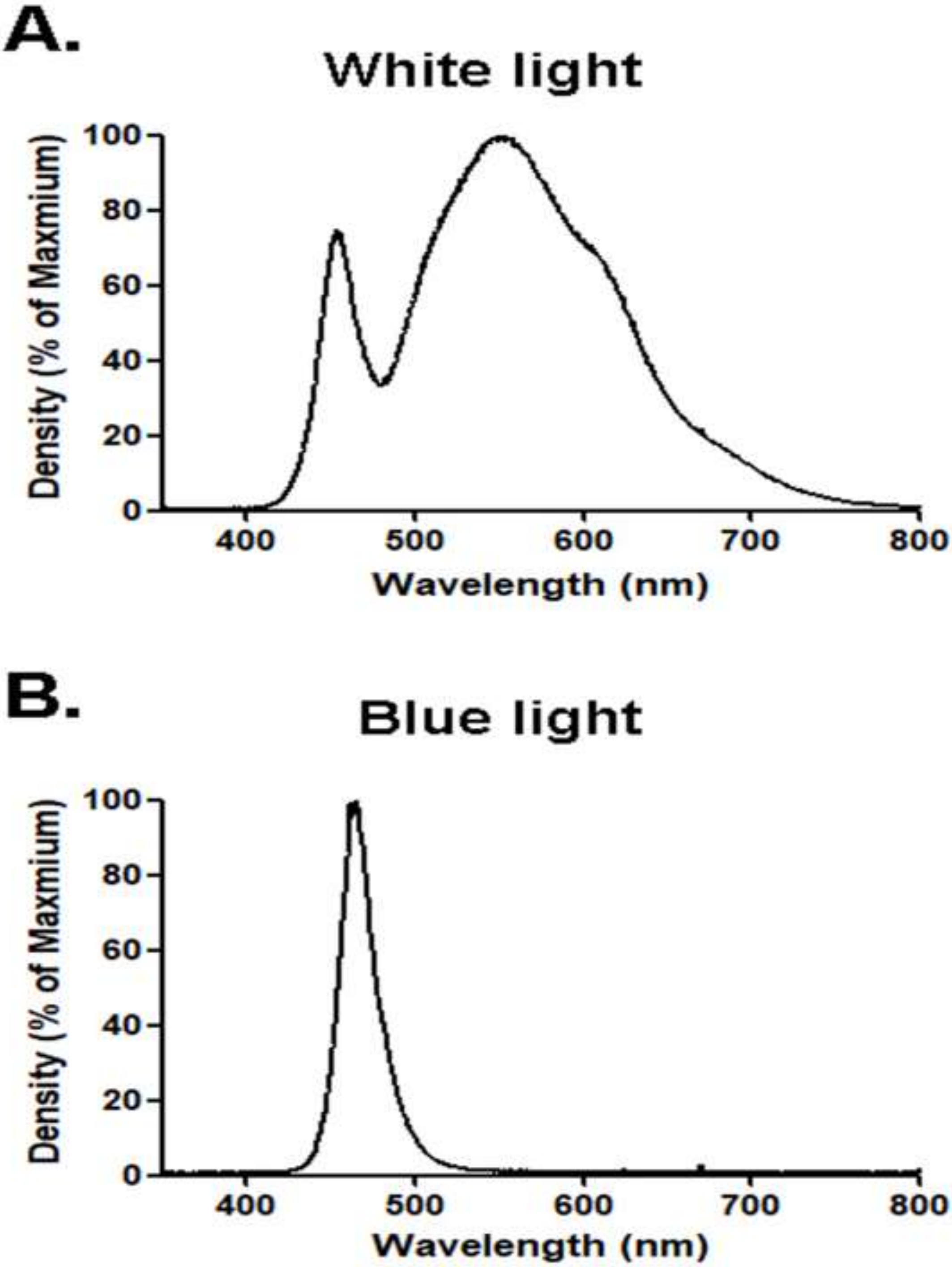
A.
White light
Density (% of Maxmium)
100
80
60
40
20
0
400
500
600
700
800
Wavelength (nm)
B.
Blue light
Density (% of Maxmium)
100
80
60
40
20
0
400
500
600
700
800
Wavelength (nm)

Supplementary Figure S1. The wavelength spectrum of white light and blue light. The ranger of wavelength showed is from 350 nm to 800 nm. The y axis presented the light density at distinct wavelength, showing as the percent of the maximum.
